# Hormonal contraception and risk for cognitive impairment or Alzheimer's disease and related dementias in young women: a scoping review of the evidence

**DOI:** 10.3389/fgwh.2023.1289096

**Published:** 2023-11-13

**Authors:** Sarah Gregory, Laura Booi, Natalie Jenkins, Katie Bridgeman, Graciela Muniz-Terrera, Francesca R. Farina

**Affiliations:** ^1^ Edinburgh Dementia Prevention, Centre for Clinical Brain Sciences, University of Edinburgh, Edinburgh, United Kingdom; ^2^ Memory and Aging Center, Global Brain Health Institute, Trinity College, Dublin, Ireland; ^3^ Centre for Dementia Research, School of Health, Leeds Beckett University, Leeds, United Kingdom; ^4^ School of Neuroscience and Psychology, University of Glasgow, Glasgow, United Kingdom; ^5^ Ohio University Heritage College of Osteopathic Medicine, Ohio University, Athens, OH, United States; ^6^ Department of Medical Social Sciences, Feinberg School of Medicine, Northwestern University, Chicago, IL, United States

**Keywords:** hormonal contraception, oral contraception, brain health, Alzheimer’s disease, scoping review

## Abstract

**Introduction:**

Women are significantly more likely to develop Alzheimer's disease and related dementias (ADRD) than men. Suggestions to explain the sex differences in dementia incidence have included the influence of sex hormones with little attention paid to date as to the effect of hormonal contraception on brain health. The aim of this scoping review is to evaluate the current evidence base for associations between hormonal contraceptive use by women and non-binary people in early adulthood and brain health outcomes.

**Methods:**

A literature search was conducted using EMBASE, Medline and Google Scholar, using the keywords “hormonal contraception” OR “contraception” OR “contraceptive” AND “Alzheimer*” OR “Brain Health” OR “Dementia”.

**Results:**

Eleven papers were identified for inclusion in the narrative synthesis. Studies recruited participants from the UK, USA, China, South Korea and Indonesia. Studies included data from women who were post-menopausal with retrospective data collection, with only one study contemporaneously collecting data from participants during the period of hormonal contraceptive use. Studies reported associations between hormonal contraceptive use and a lower risk of ADRD, particularly Alzheimer's disease (AD), better cognition and larger grey matter volume. Some studies reported stronger associations with longer duration of hormonal contraceptive use, however, results were inconsistent. Four studies reported no significant associations between hormonal contraceptive use and measures of brain health, including brain age on MRI scans and risk of AD diagnosis.

**Discussion:**

Further research is needed on young adults taking hormonal contraceptives, on different types of hormonal contraceptives (other than oral) and to explore intersections between sex, gender, race and ethnicity.

**Systematic Review Registration:**

https://doi.org/10.17605/OSF.IO/MVX63, identifier: OSF.io: 10.17605/OSF.IO/MVX63

## 1. Introduction

Sex and gender have long been recognized as important influencing factors for Alzheimer's disease and related dementias (ADRD). The lifetime risk for AD at age 45 is estimated at 1-in-5 for women and 1-in-10 for men (1). Female sex is associated with faster hippocampal atrophy (2) and greater pathological phosphorylated tau burden, key hallmarks of AD (3–5). The *APOEɛ4* gene also confers a greater risk of AD in women compared to men (at least in White populations) (6, 7). Sex hormones may explain some of the differences in risk for ADRD. Indeed, testosterone levels are a potential modifier of tau that may contribute to lower disease burden in males (3). Life-course evidence suggests pregnancy, adverse pregnancy outcomes, age of menarche, cumulative oestrogen exposure and menopause may all have implications for ADRD risk (8, 9). The potential for these biologically driven mechanisms to explain the difference in dementia prevalence by sex justifies the application of a women's health lens to the study of brain health (10).

A number of studies have investigated associations between the use of hormone replacement therapy (HRT) and brain health (9, 11–15). Comparatively less attention has been paid to the associations with hormonal contraception (HC) use. HCs act to simultaneously reduce endogenous sex hormones whilst supplementing synthetic oestrogen and/or progestin. Understanding the links between HC use and cognitive function is of considerable interest given the widespread and long-term use. Globally, over 60% of reproductive-age women use contraceptives, most of which are hormonal methods (16). The average length of time for HC use is five years, although many individuals stop and restart use across their lifespan (17). In addition to avoiding pregnancy, women use HCs for other reasons such as managing a medical condition, such as endometriosis-related pain and menorrhagia (18, 19). A recent study of young transgender individuals assigned-female-at-birth found that 80% were current or previous HC users (20, 21), highlighting the need for studies to be inclusive of this population.

The first use of HCs often occurs in young adulthood (22), a period increasingly acknowledged as a neglected stage in ADRD life-course research (23). Exposure to, and accumulation of, many modifiable risk factors (e.g., alcohol use and mental disorders 24, 25) begins during this life stage. HC use in young adulthood has been linked to changes in functional connectivity, profiled by increases in prefrontal connectivity and decreases in parietal connectivity (26). Studies have also reported changes in areas involved in affective and cognitive processing [e.g., amygdala, hippocampus, and cingulate gyrus (27)]. However, most studies were reported to have major methodological limitations regarding internal validity (27). In addition, most studies focused on short-term exposure to HCs in samples with large age ranges (i.e., 18–45 years 28). Thus, there is a need for further investigation of long-term use, especially regarding young women. Behaviourally, effects of hormone use have been reported in cognitive tasks in women (e.g., mental rotation 29). HC use is also correlated with a first diagnosis of depression, which is a known risk factor for ADRD (30).

The aim of this scoping review was to evaluate the current evidence base for associations between HC use by women, non-binary and transgender people in early adulthood and brain health outcomes.

## 2. Methods

### 2.1. Study design

A scoping review methodology was adopted to answer the research question, with a need to identify gaps in the current evidence base (31). A multi-stage approach was taken in line with scoping review methodology; define the research question, apply the PCC framework [as per Joanna Briggs Institute recommendations, the PCC (population, concept, context) framework was used to design the parameters of the scoping review (31)]; identify the databases and search terms and run the search; screen the papers; extract the data; synthesise the findings.

### 2.2. Population, concept, context

The concept was exposure to HCs. Method of action and mode of delivery included any contraceptive method classified as hormonal and targeted female reproductive systems. This included oral contraceptive (OC) pills, hormonal intrauterine devices (IUD), implant, injection, vaginal rings, and skin patches. Methods of HC were included if they contained oestrogen and/or progestin. The population was identified as female participants (where sex is reported) or women, non-binary, and transgender participants (where gender is reported) who had provided data on use of HCs. The context was selected as cross-sectional or cohort studies. Only studies that reported on the direct associations between HC use and one of the outcomes of interest (risk for dementia, cognitive impairment, other brain health outcomes related to neurodegeneration) were included. No studies that reported on indirect associations (i.e., HC use to depression to neurodegeneration) were included.

### 2.3. Databases and search terms

A literature search was conducted using EMBASE, Medline and Google Scholar, using the keywords “hormonal contraception” OR “contraception” OR “contraceptive” AND “Alzheimer*” OR “Brain Health” OR “Dementia”. Additionally, the following search parameters were added to identify any papers that additionally considered the role of gender in this topic: “women” OR “female” OR “transgender” OR “non-binary”. Papers were included if they were written in English or Spanish. No limitations were placed on the year of publication.

### 2.4. Eligibility criteria and selection

Articles were selected for inclusion in the scoping review if they reported on associations between HC use and brain health outcomes associated with ADRD or risk for ADRD. Although originally designed to only include studies reporting HC use between the ages of 18 to 39, no studies provided sufficient detail to determine this. As the majority of HC use is known to be during this age period (16), reported HC use is assumed to have been during this life stage in the included papers. A single reviewer (SG) assessed eligibility for inclusion, with 10% of papers cross-checked by a second author (KW), as recommended by Mak and Thomas (2022) (32).

### 2.5. Data extraction

A data extraction tool was created and piloted prior to use. Data extracted included (where provided) paper title, authors, year of publication, number of participants included, sex/gender breakdown, mean age of participants, HC type, duration of HC use, age started/stopped, brain health outcome measure used, and study results.

### 2.6. Narrative synthesis

A narrative synthesis was used to collate aims, methods and results across the included studies (33). The analysis involved synthesizing and summarizing findings for each outcome identified in the literature. Although we had planned to additionally synthesize results by HC type, most studies reported solely on OC use with the remaining studies providing insufficient detail to determine HC type. We reported effect estimates from studies where available (e.g., hazard ratios with 95% confidence intervals). As a final step, we outlined the broader implications for ADRD risk reduction and prevention, as well as suggestions for future studies. The scoping review was pre-registered on OSF.io (34).

## 3. Results

A total of 392 papers were identified in the initial search, 381 were not suitable after title and abstract screening with 11 papers included in the narrative synthesis. There was 100% concordance between the lead and secondary reviewer at both screening stages. Studies recruited participants from the UK (35–38), USA (39, 40), Italy (41), Indonesia (42), Singapore (43) and South Korea (13, 44). All studies except one included retrospectively collected data from women who were post-menopausal at the time of study enrolment, with only one study recruiting participants reporting on use during at early adulthood (see Table 1 for further information). No studies reported on the inclusion of participants who were non-binary or transgender, and as such the results reported relate only to papers that reported on “women” or “female participants”. The sample sizes ranged from 99 (40) to 4,696,633 participants (44). Seven studies included in the narrative synthesis reported positive associations between HC use and better brain health (13, 35, 36, 39, 40, 43, 44), whereas four studies reported no significant associations between HC contraceptive use and brain health (37, 38, 41, 42).

**Table 1:** Table of papers included in narrative synthesis.

Paper	Participants	Hormonal contraception	Menopausal status	Brain health outcome	Results
Associations between hormonal contraception and ADRD risk					
Gong et al. (35).	UK Biobank, 273,240 women and 228,957 men. No information on the inclusion of non-binary or transgender individuals. Mean age of women was 56 years. 94.2% women of White ethnicity; 5.8% women of other ethnicity. Country: UK	Use of OC and age of initiation. No information available on type of OC or dosage. Data was retrospectively collected via self-report.	61% of women included self-reported being postmenopausal by natural menopause (mean age at natural menopause 50.3 years). 19% of women included self-reported having a hysterectomy (mean age 43.9 at hysterectomy years). 8% of women self-reported having an oophorectomy (mean age at oophorectomy 47.4 years).	Incident all-cause dementia.	HR for dementia in those who reported oral contraceptive use was 0.80 (95% CI: 0.72, 0.88), *p* < 0.001. No association with age of starting use of OC. Lower risk only statistically significant in women younger than 65 years at study baseline.
Song et al. (43).	Singapore Chinese Health Study, 8,222 post-menopausal women. No information on the inclusion of non-binary or transgender individuals. Mean age 53.4 years. All women Singapore Chinese (49.8% Cantonese dialect speakers, 50.2% Hokkien dialect speakers). Country: Singapore.	Use of OC for at least one month and duration of use. No information available on type of OC or dosage. Data was retrospectively collected via self-report.	All women self-reported natural menopause. 6.2% menopause before 45 years, 27.9% menopause aged 45–49 years, 53.0% menopause aged 50–54 years, 12.9% menopause aged 54 years and older.	SM-MMSE to determine cognitive impairment (Cut off points determined appropriate to local population; no education: 17/18; primary school education: 20/21; secondary school or more: 24/25).	Women with ≤5 years of OC use had a lower risk of cognitive impairment compared to those who had never used OC HR: 0.74 (95% CI: 0.63, 0.87). Not statistically significant for >5 years (HR 0.87 (95% CI: 0.68, 1.13).
Yoo et al. (44).	Korean National Health Insurance System, 4,696,633 post-menopausal women. No information on the inclusion of non-binary or transgender individuals. Mean age 61.2 years. No information on race or ethnicity available. County: South Korea.	Use of OC. No information available on type of OC or dosage. Data was retrospectively collected via self-report.	Menopausal status self-reported via questionnaire, participants with hysterectomy procedure in general excluded (*n* = 17,667). 1.6% of participants reported menopause prior to 40 years, 5.3% menopause between 40 and 44 years, 25.9% menopaused between 45 and 49 years, 55.4% menopause between 50 and 54 years, 11.8% menopause aged 55 years and older.	Diagnosis of dementia.	Use of OC reduced the dementia risk by 10%, with no differences seen in duration of use: <1 year use HR: 0.91 (95% CI: 0.88, 0.92); ≥1 year use HR: 0.90 (95% CI: 0.88–0.92).
Kim et al. (13).	Korean National Health Insurance System, 209,588 post-menopausal women. No information on the inclusion of non-binary or transgender individuals. Mean age 61.5 years non-dementia group (*n* = 179,723), 70.46 years in dementia group (*n* = −29,865). No information on race or ethnicity available. County: South Korea.	Lifetime use of OC (“never,” “use for less than 1 year,” “use for more than 1 year,” or “unknown.”). No information available on type of OC or dosage. Data was retrospectively collected via self-report.	Menopausal status self-reported via questionnaire, participants with history of hysterectomy excluded (*n* = 324,425). Mean age at menopause 49.99 years in non-dementia group, 49.27 years in dementia group.	Diagnosis of dementia, including sub-type analysis (Alzheimer's disease dementia: ADD; vascular dementia: VD).	Dementia (all-cause): OC use <1 year HR: 0.92 (0.88, 0.96). OC use ≥ 1 year HR>: 0.90 (0.86, 0.95). ADD: OC use <1 year HR 0.92 (0.88, 0.97); OC use for ≥1 year HR 0.89 (0.84–0.94). VD: OC use <1 year use HR: 0.96 (95% CI: 0.84, 1.10); OC use ≥1 year use HR: 0.97 (95% CI: 0.84, 1.13)).
Fox et al. (38).	89 women aged 70–100 years. No information on the inclusion of non-binary or transgender individuals. Median age 77 years in control group (*n* = 51), 86 years in the patient group (*n* = 38). All participants were White British, living in England. Country: UK.	OC use. No information available on type of OC or dosage. Data was retrospectively collected via self-report.	Age of experiencing menopause self-reported via interview. Median age at menopause 50 years in control group, 50 years in patient group.	Age at Alzheimer's onset.	No association between OC use and AD risk.
Zucchella et al. (41).	Case control study, 275 women with AD and 276 controls. No information on the inclusion of non-binary or transgender individuals. Mean age 77.6 years in AD patient group, 76.7 years in control group. No information on race or ethnicity. Country: Italy.	History of at least 6 months of HC use. No information available on type of OC or dosage. Data was retrospectively collected via self-report.	Menopause type (physiological or surgical) and age of menopause self-reported via interview. 89% of AD patients and 84.1% of controls had a physiological menopsause. 11% of AD patients and 15.9% of controls hd a surgical menopause.	ADD vs. control.	No differences between groups in history of OC use (*χ*^2^: 1.61, *p*: 0.20).
Pradono et al. (42).	Prospective cohort study of 2,668 female participants. No information on the inclusion of non-binary or transgender individuals. Mean age 47.4 years. No information on race or ethnicity. Country: Indonesia.	Data on HC use (Yes/No) collected. No information available on type of OC or dosage. Data on current use was collected via self-report.	No information on menopausal status of participants. 2.5% of participants reported using HRT.	Subjective memory complaint based on a positive response to the question: Are you considered forgetful by others (family, friends, etc)?	No significant associations between HC use and with subjective memory complaints in fully adjusted models [OR: 1.30 (95% CI: 0.995, 1.70)].
Associations between hormonal contraception and cognition					
Lindseth et al. (36).	UK Biobank, 221,124 women. No information on the inclusion of non-binary or transgender individuals. Mean age 56.2 years. 95.2%White, 1.5% Asian, 1.5% Black, 0.4% Chinese, 0.8% Other ethnic groups, 0.6% mixed ethnicity. Country: UK.	HC usage (current user, past user, never user) and duration of use and age of initiation. No information available on type of OC or dosage. Data was retrospectively collected via self-report.	75% of participants reported a natural menopause (mean age at natural menopause 50.5 years). 3% of women included self-reported having a hysterectomy (mean age 44.8 at hysterectomy years). 3% of women self-reported having an oophorectomy (mean age at oophorectomy 50.2 years).	Computerised cognitive tests assessing visual memory, working memory, processing speed and executive function.	Both current and past use of HC were significantly associated with higher scores on all cognitive tasks. Longer duration of hormonal contraceptive use was associated with higher performance on all tasks except visual memory. Older age at initiation was associated with lower performance in all cognitive tests.
Egan and Gleason (39).	Wisconsin Registry for Alzheimer's Prevention, 261 women. No information on the inclusion of non-binary or transgender individuals. Mean age 52 years. 98.2% of previous HC users were White, 0.9% Black, 0.4% American Indian and 0.4% Hispanic, whilst 100% of never users were White. Country: USA.	HC use history and duration. No information available on type of OC or dosage. Data was retrospectively collected via self-report.	78% of the previous HC use group self-reported as postmenopausal (no age of menopause available), whilst 64.8% of the never HC use self-reported as post-menopausal.	17 cognitive tests combined into 5 domains: Verbal Ability, Visuo-spatial Ability, Working Memory, Verbal Learning & Memory, and Speed & Flexibility.	Use of HC associated with better visuospatial ability [mean difference: 0.75 (95% CI: 0.23, 1.28)] and speed and flexibility [mean difference: 0.52 (95% CI: 0.14, 0.90), *p*: 0.007]) compared to those who had never used, with strongest effects seen in those with ≥15 years of use.
Associations between hormonal contraception and MRI measures					
Schelbaum et al. (40).	99 women and 26 men,. No information on the inclusion of non-binary or transgender individuals. Women: Mean age 52 years. Women: 80% White, 6% Asian, 6% Black/African America, 6% Mixed, 4% Hispanic. Country: USA.	Use of HC and duration of use. No information available on type of OC or dosage. Data was retrospectively collected via self-report.	Menopausal status defined by Staging of Reproductive Aging Workshop criteria and laboratory hormone assessments, menopause type categorised as spontaneous or induced. 50% were post-menopausal (74% spontaneous, 26% induced). Mean age at menopause 51 years.	Cognitive tests (verbal memory, executive function, language). MRI parameters (grey matter, white matter).	Positive associations between HC use and grey matter volume were observed in precuneus, fusiform gyrus, superior parietal lobule, angular gyrus, and inferior frontal gyrus of the left hemisphere and in fusiform gyrus of the right hemisphere (all *p* < 0.005). No associations with cognition.
de Lange et al. (37).	UK Biobank, 16,854. No information on the inclusion of non-binary or transgender individuals. Mean age 54.7 years. 97.4% White, 0.7% Asian, 0.6% Black, 0.5% Mixed, 0.5% Other, 0.3% Chinese. Country: UK.	Use of OC and age of initiation. No information available on type of OC or dosage. Data was retrospectively collected via self-report.	50.97% self-reported they had had their menopause, 30.29% reported they had not had their menopause, 21% were not sure and 0.08% preferred not to answer. Amongst HRT users the mean age at menopause was 48.5 years, and amongst non-HRT users the mean age at menopause was 50.6 years.	Brain age.	No significant association between OC status and brain age (*β*: 0.02, SE: 0.07, p*_corr_*: 0.80).

AD, Alzheimer's disease; ADD, Alzheimer's disease dementia; CI, confidence interval; HC, hormonal contraceptive; HR, hazard ratio; HRT, hormone replacement therapy; MRI, magnetic resonance imaging; OC, oral contraceptive.

### 3.1. Associations between hormonal contraception and ADRD risk

Four studies reported associations between HC use and a lower risk of ADRD (35, 43, 44), particularly AD (13), whilst three studies found no significant associations between risk of AD diagnosis (38, 41) and subjective memory complaints (42).

In a study of UK Biobank participants, 81% of women reported using OCs. In this population, OC use was associated with a reduced risk of dementia with no evidence of an association between age of first use of OCs and risk of dementia (35). Subgroup analysis identified this association was only seen in women below 65 years of age at study baseline, suggesting OC use may only confer a benefit until a particular stage of life. A study of women from the Singaporean Chinese population similarly found 81% of participants had previously used OCs, with the majority reporting less than 5 years of use. Those who used OCs for less than 5 years were found to have a reduced risk of dementia compared to those who had never used OC, but interestingly, this association was not seen in those with more than 5 years of use (43). In contrast, a study utilising data from the South Korean NHIS found most women (80.6%) had never used OCs. Despite this, the analysis found OC use was significantly associated with approximately 10% lower risk of dementia compared to those who had never used OCs, with no differences between less than or more than a year's use (44).

A second study using both the South Korean NHIS and the National Cancer Screening Programme investigated subtypes of dementia. This study reported similarly low use of OCs (16.4% documented use) and found similar reductions in risk for all-cause dementia. Considering subtypes of dementia there remained a significant association between OC use and decreased risk of AD, however, no significant association with risk for vascular dementia (13).

This specific association between OC use and risk for AD has not been replicated in other studies. In a British cohort study with low OC use rates (26% previous or current users), there was no significant association with the diagnosis of AD (38). This study included a comparatively small sample size with low rates of OC use compared to rates reported in the UK Biobank cohort which limits confidence in interpreting these results. Participants included in this study were recruited between the ages of 70–100 years, therefore many participants included would not have had access to HCs during their early adulthood explaining the low usage rates reported. Another case-control study recruiting participants up to their 9th decade of life in Italy with similarly low rates of HC use (3% previous use in patient group, 5% previous use in control group), found no significant associations between HC use and diagnosis of AD (41). Finally, a study in Indonesia recruiting women aged 25 and above (mean ∼47 years) with significantly higher HC usage (72% reported use) found no significant association with subjective memory complaint cases (42).

### 3.2. Associations between hormonal contraception and cognition

Two studies reported associations between HC use and better performance on cognitive tasks. In a study using the UK Biobank (current HC: 2%, previous HC: 78%), both past and current HC use was associated with significantly higher test scores on tasks measuring processing speed, executive functioning, and visual and working memory. Longer duration of HC use was also associated with better performance on most cognitive tests, whilst an older age of starting HC use was associated with lower performance across all cognitive domains (36). A second study recruiting participants from the USA, with similar high HC usage rates (87% current or previous users), found significantly higher performance on tasks of visual-spatial ability and speed. There were no significant differences by HC use on tasks of verbal ability, working memory and verbal learning and memory (39).

### 3.3. Associations between hormonal contraception and MRI measures

Only two studies have reported on associations between HCs and MRI measures, with mixed results. A study recruiting participants in the USA (9% current users, 53% past users) reported significant associations between use and larger grey matter volume in the precuneus, fusiform gyrus, superior parietal lobule, angular gyrus and the inferior frontal gyrus of the left hemisphere and in fusiform gyrus of the right hemisphere (40).

A UK Biobank data analysis (86% HC users) found no association between usage and brain age (37). No other studies were identified that reported MRI outcomes and HC use in the context of ADRDs.

## 4. Discussion

Of the eleven papers included in this narrative review, the majority investigated associations between HC use and risk for dementia. Studies that were typically larger and with higher rates of OC use reported significant associations with decreased risk for dementia, including AD, however smaller studies including older women with lower rates of previous OC use reported no associations with AD risk. One study recruiting women from young adulthood did not find associations between HCs and subjective memory complaints. Two studies reported significant associations with better performance on cognitive tasks with HC use, and whilst one study reported higher grey matter volume amongst HC users, a second MRI study found no association with brain age. There is clearly a need for more research on this topic, with a particular need to focus on data collection within the age group most likely to be using HCs (young adults), more detailed investigation by HC type and an expansion of outcomes of interest to include more specific research around associations with cognitive performance and brain MRI measures relevant to ADRDs.

Studies conducted to date focus exclusively on associations between OCs and brain health or did not define what was meant by HC within their database used, with no studies explicitly considering other HCs such as the implant, injection or hormonal IUD. Understanding the associations between HCs and brain health is critical, as these methods continue to grow in popularity and may exert more localised effects compared to oral contraception. For example, one hormonal IUD has been associated with increased stress reactivity (45), demonstrating the potential for side effects of the contraception outside of the localised effects, and may raise important implications for ADRD given known associations with cortisol (46).

Another potential limitation that must be addressed is the reliance on retrospective data collection, which may be less accurate due to self-reporting events in the past that may not be recalled accurately, leading to potential recall bias (47). Future studies should collect data from participants in early adulthood to understand whether the potential benefits for brain health and ADRD risk reduction can be seen throughout the lifespan. There are also inherent limitations in all observational studies in establishing a direct cause-effect relationship which should be acknowledged. It is also important to consider confounding by indication, such as psychological factors, sexual debut or abstinence and personality that lead to decision to use HCs which may themselves be determined by brain function or structure (27). In addition, the menopausal status of participants included in the individual studies may have influenced the results independent of previous HC use, given known associations between menopause and brain health (48) (including importantly the potentially reversible nature of brain fog experienced in peri-menopause identified in the SWAN studies that may have otherwise influenced cognitive performance in some of these studies 49). Understanding whether exposure to exogenous and synthetic hormones (both as contraceptives and HRT) can modify the risk for future dementia will be important, as evidenced by a recent paper from the UK Biobank which reported significant associations between more prolonged exposure to endogenous hormones and smaller burden of cerebral small vessel disease independent of HC and HRT use (50). Conversely, a study in Sweden found that a longer reproductive period was associated with a higher risk for future dementia, again independent of HC use (51). Slight differences in reproductive periods between the two studies discussed here (37 vs. 34 years on average) as well as different outcomes (small vessel disease on MRI vs. dementia diagnosis) may explain these findings, and highlights the need to develop a more comprehensive literature base in this area to understand the role of HCs, HRT, menopause and lifetime exposure to endogenous sex hormones in relation to brain health and risk for neurodegeneration. Future research could also focus on exploring associations between HC use and more novel and sensitive markers of neurodegeneration, including the use of amyloid and tau positron emission tomography (PET) scans, cerebrospinal fluid (CSF) analysis and blood-based biomarkers (52, 53). As previous research has focused on global cognitive assessments, or the diagnosis of dementia, this would provide evidence on any associations between HCs and the earliest stages of AD in particular, which could help to delineate whether there is an optimal time window where exogenous hormones may confer brain health benefits.

All studies focused exclusively on the dichotomy of sex and did not consider whether gender is relevant to this discussion. Even where studies report on “sex”, there are often no documented definitions for these categories, and as such participants may have chosen whether to self-report sex or gender (54). There is also emerging evidence that there are changes on the brain throughout the menstrual cycle, as demonstrated by a 1.3-year decrease in brain age at ovulation compared to other times (55). This highlights the critical need to consider the role of sex and gender, as well as the use of exogenous hormones, throughout the spectrum of ADRDs, from prevention to detection and treatment. Similarly, studies did not consider race and ethnicity, even though Black women are at the highest risk for ADRD (56). These will be important demographic data points for future studies to consider. Research in ADRD risk factors has long espoused inequitable population representation within their participants, with minority and socioeconomically disadvantaged individuals being underrepresented (57). The research community has typically attributed the under-representation to such groups being naturally “hard to reach” and more challenging to involve (58). A key lesson from previous work is that it is more often the researchers' approach and attitude to engagement that restricts the diversity of public involvement, rather than the enthusiasm and interest of the public concerned (59). Further efforts are needed to systematically evaluate approaches that successfully foster equitable involvement and engagement in research recruitment and retention, including improved methodological standards such as Public and Participant Engagement and Involvement (PPIE) with a focus on under-served populations (60) and involvement with the voluntary and community sector enterprises who support these populations.

An important avenue for future research will be to examine indirect pathways between HCs and ADRD risk, including their impact on modifiable risk factors for ADRD. The Lancet Commission for dementia prevention, intervention, and care lists 12 modifiable risk factors for dementia including hypertension, traumatic brain injury (TBI), depression and diabetes, all of which are affected by HC use (61). For example, HCs increase blood pressure in the majority of women, 5% of whom will develop hypertension (62). Hypertension confers a 2% population attributable fraction (PAF) in high income countries (61), and between 4 and 8% PAF in low- and middle-income countries (LMICs) (63) according to the Lancet Commission, and as such the potential for this indirect association warrants further investigation. Conversely, it has been demonstrated that HCs may reduce the severity of symptoms and duration of recovery following TBI (64). How these indirect influences on known modifiable risk factors affect dementia risk is unknown with more research needed. Given the heterogeneity of women using HCs it is important to consider that associations between HCs and brain health may vary within this group, it is possible that there are subgroups of women where ADRD risk is not reduced and may in fact be increased. It may also be important to consider any interactions between the *APOEe4* gene, HC use and brain health outcomes, given evidence suggesting this gene mediates associations between HRT and cognitive impairment (11). One of the papers included in this scoping review did look at this and reported no interaction between *APOEe4* HC use and cognition (39), with no other studies reporting on this.

Though limited, most studies identified here suggested a positive association between HC use and brain health. The possibility that HCs can confer brain health benefits has significant real-world implications and challenges. Despite efforts to improve women's health, disparities in access and utilization of reproductive services continue to persist. Inequalities have been documented across racial, ethnic, and socioeconomic groups, as well as across geographical regions (65–67). A recent Lancet systematic analysis reported that over 160 million women had an unmet need for contraception in 2019 (67); most of these women resided in sub-Saharan Africa and South Asia. Young women aged 15–24 years had the lowest rates of demand satisfaction. Optimizing any potential benefits of HCs on brain health will therefore require significant efforts to reduce inequities in access and utilization.

In summary, there is a small but growing evidence base suggestive of potential brain health benefits of HCs for women. Further work is needed to address current limitations and work directly with the age group most likely to take these contraceptives, with the goal of understanding if and how these may be used as a tool in ADRD risk reduction and prevention efforts.

## Data Availability

The original contributions presented in the study are included in the article/Supplementary Material, further inquiries can be directed to the corresponding author.
